# Multimorbidity risk assessment in adolescents and adults with cerebral palsy: a protocol for establishing a core outcome set for clinical research and practice

**DOI:** 10.1186/s13063-019-3265-z

**Published:** 2019-03-19

**Authors:** Patrick G. McPhee, Joyce L. Benner, Astrid C. J. Balemans, Olaf Verschuren, Rita J. G. van den Berg-Emons, Edward A. Hurvitz, Mark D. Peterson, Wilma M. A. van der Slot, Marij E. Roebroeck, Jan Willem Gorter

**Affiliations:** 1 School of Rehabilitation Science, McMaster University, Institute for Applied Health Sciences, 1400 Main Street West, Hamilton, ON L8S 1C7 Canada; 2 Department of Rehabilitation Medicine, Erasmus University Medical Center and Rijndam Rehabilitation, P.O. Box 2040, 3000 CA Rotterdam, The Netherlands; 3 Brain Center Rudolf Magnus and Center of Excellence for Rehabilitation Medicine, University Medical Center Utrecht and De Hoogstraat Rehabilitation, Rembrandtkade 10, 3583 TM Utrecht, The Netherlands; 4 Department of Rehabilitation Medicine, Amsterdam Movement Sciences, Amsterdam Public Health, VU University Medical Center, Amsterdam, The Netherlands; 5 Department of Physical Medicine and Rehabilitation, University of Michigan, 325 E Eisenhower Parkway, Ann Arbor, MI 48108 USA; 6 Department of Pediatrics, McMaster University, Institute for Applied Health Sciences, 1400 Main Street West, Hamilton, ON L8S 1C7 Canada

**Keywords:** Cerebral palsy, Adolescents, Adults, Health, Core outcome set, Multimorbidity, COMET, COSMIN

## Abstract

**Background:**

Estimates of multimorbidity, defined as the presence of at least two chronic conditions, some of which attributable to modifiable behaviours, are high in adults with cerebral palsy (CP). An assessment protocol evaluating multimorbidity risk is needed in order to develop and evaluate effective interventions to optimize lifelong health in individuals with CP. The aim of this protocol paper is to describe the development of a core outcome set (COS) for assessing multimorbidity risk in adolescents and adults with CP, to be used in clinic and research.

**Methods:**

The expert consortium will first define the target population and outcomes to be measured. Through a process of literature review and an international Delphi survey with expert clinicians and researchers, we will then determine which outcome measurement instruments (OMIs) can best measure those outcomes. The resulting OMIs will be used in a feasibility study with adolescents and adults with CP from an international clinical research network. Finally, a face-to-face stakeholder meeting with adolescents and adults with CP, their families/caregivers and researchers and clinicians who are experts in CP, will be organized to reach final agreement on the COS.

**Discussion:**

This COS will guide clinicians and researchers in assessing multimorbidity risk in adolescents and adults with CP. The inclusion of experts and individuals with CP from international locations for establishing the COS lends strong support to its generalizability. Evidence of its feasibility and approval from all stakeholders will enable implementation in clinical practice, and guide future research using the COS in individuals with CP.

**Electronic supplementary material:**

The online version of this article (10.1186/s13063-019-3265-z) contains supplementary material, which is available to authorized users.

## Background

Cerebral palsy (CP) is a well-recognized neurodevelopmental disability commencing in early childhood and continuing throughout life, and is the most common motor disability in childhood [1]. The disability itself results from non-progressive disturbances to the developing fetal or infant brain, and the resultant motor disorders are often accompanied by disturbances of cognition, behaviour and communication, to name only a few [2]. Population-based studies report prevalence estimates of CP ranging from 1.5 to greater than 3 per 1000 live births [3–7]. As CP presents itself early in life, much research has focused on children with CP; however, given the longer lifespan apparent in most persons with CP [8], clinicians and researchers have started to focus on the impact of CP and associated health issues using a lifespan approach. Indeed, adults with CP are a growing community who are now recognised as outnumbering children 3:1 in some countries [9].

A prominent concern for individuals with CP is their physical behaviour and reduced cardiorespiratory endurance [10–12]. Physical behaviour is defined as the behaviour of a person in terms of body posture (e.g. sitting and standing), movements (e.g. walking and cycling) and/or daily activities (e.g. sports and gardening) in his/her own environment, and therefore consists of both physical activity and sedentary behaviour [13]. Cardiorespiratory endurance is the capacity of the body to perform physical activity, which is dependent mainly on the aerobic or oxygen-requiring energy systems [10]. Adolescents and adults with CP have reduced cardiorespiratory endurance [14], which is a risk factor for cardiovascular disease and cardiovascular-related mortality [11]. Also, children, adolescents and adults with CP engage in significantly less physical activity and more sedentary behaviour compared to typically developing peers [15–19]. Low levels of physical activity and more sedentary behaviour can partly be explained by reduced mobility following from the condition itself, and by accompanying physical pain and fatigue that progressively worsen with aging [20]. There are differences among individuals with CP in their physical activity levels [21] and in the prevalence of obesity [22], which are contingent upon the functional status of individuals, as determined by the Gross Motor Function Classification System (GMFCS) [23]. Lower cardiorespiratory endurance and physical activity and more sedentary behaviour are associated with risk of cardiovascular disease (i.e. coronary heart disease, cerebrovascular disease, peripheral arterial disease) and cardiometabolic disease (i.e. diabetes mellitus and obesity) in persons with CP [17, 24, 25], which may become higher later in life [26, 27]. Recent research in middle-aged adults with CP revealed high estimates of multimorbidity, which were significantly more prevalent among obese versus non-obese persons with CP [12].

Multimorbidity has been defined as the presence of at least two chronic conditions [12]. Among individuals with CP, reports are emerging of chronic conditions apart from CP itself, such as hypertension, dyslipidaemia, hyperglycaemia, insulin resistance and obesity [17, 24, 26]. Results from a population-representative sample of adults with CP showed that this population has significantly greater age-adjusted prevalence of hypertension (30.0% vs. 22.1%) and obesity (41.4% vs. 29.7%) compared to adults without CP [28]. Despite the significant progression of disability that is known to occur during the aging process in CP [20], there has been a lack of attention devoted to understanding the pathophysiology of the development of multimorbid conditions in adolescents and adults with CP, beyond those stemming from the primary brain injury in infancy. Risk of multimorbidity could be attributed to a shared number of modifiable behaviours such as physical inactivity and/or sedentary lifestyles, poor diet and inadequate sleep [29]. This highlights the importance of screening for, and understanding of exposure to factors that increase the risk of multimorbidity in individuals with CP.

Over the last decade, a number of generic and CP-specific instruments and protocols assessing multimorbidity risk factors have been developed. As a result, studies evaluating these risk factors in adolescents and adults with CP are using a variety of outcome measurement instruments (OMIs) (e.g. self-report questionnaires, accelerometry-based activity monitors, biomarkers and performance-based tests), which might be measuring the same outcome, and thus causing difficulty synthesizing knowledge from the published literature and when generalizing findings [30]. Moreover, the psychometric quality (i.e. reliability, validity, sensitivity) of OMIs tends to vary and/or published evidence is lacking, altogether making it inconvenient for clinicians and researchers to select the most appropriate OMIs for the outcome of interest. In order for clinicians and researchers to work with individuals with CP on plans for effective interventions - including advice pertaining to physical behaviour, nutrition and sleep - to reduce multimorbidity risk, it is vital to reach consensus on which outcomes to assess, the ways to assess them, and ultimately leading to their inclusion in routine clinical practice.

Lately, there is increasing recognition of the need to identify core sets of outcomes that enable comparison of clinical trials in a particular condition. Moreover, establishing a core outcome set (COS) may be useful for routine health screening. Currently, there is no established COS for adolescents and adults with CP for the purpose of evaluating multimorbidity risk factors. A search of “cerebral palsy” through the Core Outcome Measures in Effectiveness Trials (COMET) database resulted in six matches, all of which focus on children with CP [31]. There are common data elements (CDEs) for CP due to a joint effort between the CP CDE Working Groups and the National Institute of Neurological Disorders and Stroke [32]. Within the CDEs is a summary of core and supplemental recommendations that is highly recommended as a start-up resource for clinical research in this population. Although this set of CDEs was recently developed (2016), it only applies to children and adolescents aged 0–18 years and does not specify instruments specific to adults with CP or measures that assess multimorbidity risk [33].

The aim of this protocol paper is to describe the process of developing a COS of OMIs for multimorbidity risk in adolescents and adults with CP, to be used in clinic and research. This work includes (1) identifying what outcomes should be measured; (2) determining how to best measure those outcomes and (3) measuring these outcomes in an international cohort of individuals with CP. The final COS will be made in consultation with individuals with CP and their families and caregivers and with representatives of the clinical and research community who are working with people with CP. The inclusion of adolescents and adults with CP and their families and caregivers is critical to ensure that OMIs are meaningful, appropriate, and acceptable to inform decisions about the assessment of multimorbidity risk in this population. We will include adolescents with CP in the assessments of multimorbidity risk, as this will capture a pivotal transition period, and may highlight the importance of engaging in positive behaviours early on to attenuate multimorbidity risk later in life. This study is part of a programme of research aiming to ultimately understand, treat and prevent multimorbidity in adolescents and adults with CP through modifiable behaviours (e.g. physical behaviour, sleep and nutrition), and to develop an international database that will allow for harmonization of data and the ability to document changes over time in this population.

## Methods

**Fig. 1:**
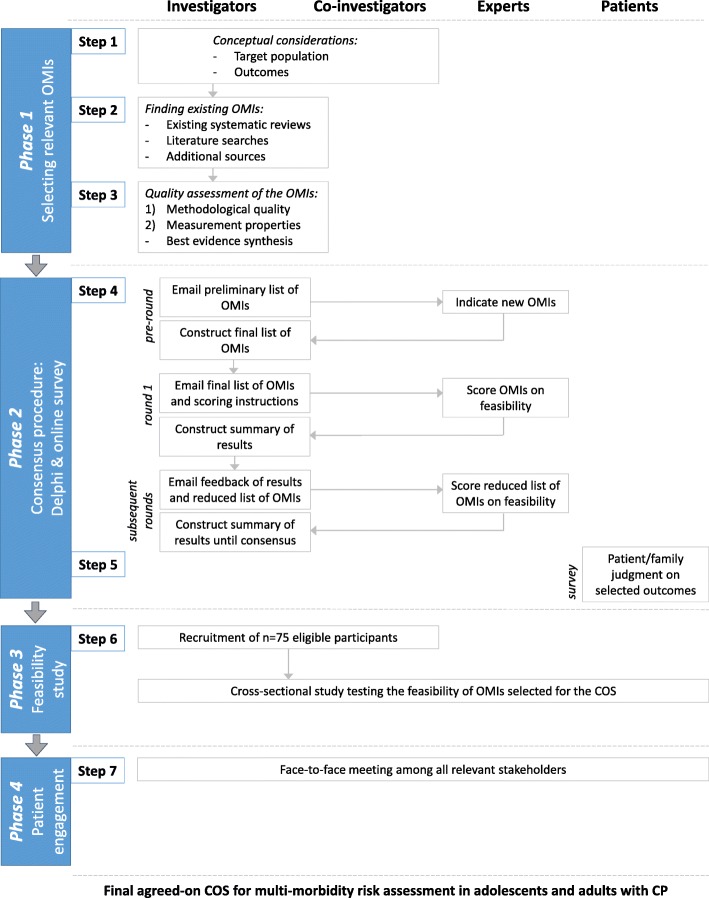
Main steps in the core outcome set (COS) development including roles of all involved at each step. Schematic outline of the different phases and steps included in the development of a COS for multimorbidity risk assessment in adolescents and adults with cerebral palsy. Roles of all involved at each step are indicated. OMI, outcome measurement instrument; CP, cerebral palsy

This study protocol is registered with the COMET Initiative (http://www.comet-initiative.org/studies/details/1130) and follows recently published guidelines from a collaboration between the COMET Initiative and Consensus-based standards for the selection of health measurement instruments (COSMIN) [30]. Slight adjustments were made to the original flowchart [30], as we chose to include a pilot testing phase (Fig. 1). Phase 1 has begun, and the target population and outcomes to be measured have already been defined.

### Investigators and co-investigators

Four authors (PM, JB, MR and JWG) will have an investigating role in the COS development. After deciding on the conceptual considerations in consultation with co-investigators (MP, EH, WS, AB, OV and RB), they will first review the literature and extract relevant OMIs. Second, they will carry out a consensus procedure with experts to obtain agreement on selected OMIs to be included in the COS. Third, they will coordinate a cross-sectional feasibility study in which the COS will be tested in an international cohort of adolescents and adults with CP at four sites. Finally, they will organize a stakeholder meeting to review and finalize the COS of OMIs for multimorbidity risk.

#### Phase 1: selecting relevant OMIs

##### Step 1: conceptual considerations

We defined a target population and outcomes to be measured, in line with the COSMIN-COMET guideline [30]. This was done via an in-person meeting with the co-investigators from four research centres (Hamilton, Canada; Ann Arbor, USA; Rotterdam and Utrecht, The Netherlands), at the 29th European Academy for Childhood Disability conference (2017). During this meeting, the target population was defined as adolescents (14–18 years of age) and/or adults (> 18 years of age) with CP. Furthermore, the following outcomes were decided to be important for assessing multimorbidity risk in the target population: physical behaviour, nutrition, sleep, cardiorespiratory endurance, body composition, blood pressure and lipids/glucose. Including risk behaviours (i.e. physical behaviour, nutrition and sleep) is important to identify which patients require intervention. Measuring cardiorespiratory endurance, body composition, blood pressure and lipids/glucose will allow clinicians and researchers to observe the benefits of improved risk behaviours.

##### Step 2: finding existing OMIs

In order to find all existing OMIs addressing the defined outcomes, we will use three sources of information, including (1) existing systematic reviews, (2) literature searches and (3) additional sources (e.g. conference proceedings) [30]. Additional sources are considered optional since it is unlikely that one will find OMIs of good quality that were not already identified from a systematic search of the literature [30].

The COSMIN database of systematic reviews of OMIs will be consulted to see if there are any systematic reviews that describe our target population and any of the seven outcomes. Candidate OMIs for each outcome will further be identified by electronic searches of the following databases: EMBASE and Medline (Ovid), PsycINFO and Pubmed. The electronic searches will be carried out by two researchers with experience in conducting systematic reviews (PM and JB). We will develop a search strategy that will include the following major themes: “cerebral palsy”, “adolescent OR adult” and each outcome on its own. Key terms within the search strategy will be aligned to medical subject headings and expanded to include more descriptive terms. Searches consistent with “measurement properties” will not be included in the search strategy, since evidence on the measurement properties of relevant OMIs is expected to be limited in our target population, exposing a risk of missing relevant studies (see Additional file 1). Eligible publications will be randomized controlled trials, longitudinal studies (including experimental and cohort studies) and observational studies (including cross-sectional, cohort, and case-control studies) written in English. Studies will be grouped by outcome and repeated where appropriate (i.e. if there is > 1 outcome within a single study). The two researchers will independently screen titles and abstracts and select references using a predetermined set of inclusion/exclusion criteria (see Additional file 2). If there are any discrepancies, these will be resolved by consulting other investigators (JWG and MR). Upon agreement on the final selection of studies, the two researchers will record each OMI for each outcome used in an eligible study. We will also extract characteristics of the study sample (i.e. sample size, mean age, sex, type of CP and GMFCS level(s)). Data extracted will be crosschecked for comparison of accuracy between the two researchers.

A group of experts that will be consulted during the second phase of the COS development process will serve as additional sources for finding OMIs. The group will be requested to provide any additional OMIs that are considered relevant to an outcome but were not identified in current systematic reviews or in the literature searches.

##### Step 3: quality assessment of the OMIs

The quality of the OMIs that result from step 2 will be assessed in accordance with the COSMIN-COMET guideline [30]. The quality assessment will include two parts: (1) evaluating the methodological quality of the studies included from the literature searches and (2) evaluating the quality of the OMIs (i.e. their measurement properties). Since the literature search will not be limited to studies on the measurement properties of OMIs, we will use a combination of the COSMIN checklist and the McMaster critical review form [34, 35]. The COSMIN checklist will be applied for evaluating the methodological quality of studies on the measurement properties of OMIs [34], while the McMaster critical review form will be applied to assess the methodological quality of the other study types (e.g. clinical trials and observational studies) [35]. The quality of the OMIs will be evaluated by applying criteria for good measurement properties [36]. We will first evaluate the content validity of the included OMIs and where applicable the remaining measurement properties [30]. Both researchers (PM and JB) will perform the OMI evaluation, and will crosscheck each other’s quality assessments to ensure accuracy and completeness. Evaluations of the methodological quality of the studies and the quality of the OMIs will be combined into a best evidence synthesis, grading the body of evidence for each OMI [30]. Feasibility aspects of the OMIs including applicability (for the target population), patient feasibility, assessor/clinician feasibility and practical feasibility will be considered in the next phase of the study.

#### Phase 2: consensus procedures: Delphi and online survey

##### Step 4: select an OMI for each outcome included in the COS

We will use the Delphi survey method [37] as a consensus procedure to obtain agreement on the selected OMIs included in developing a COS, performed by experts in the area of the risk of multimorbidity in adolescents and adults with CP. In a Delphi procedure, interactions between experts occur via a series of individual surveys, preserving both anonymity and balance in the participation of the experts [38]. In contrast to an in-person consensus method, a Delphi procedure can be conducted via email survey and is therefore accessible to participants regardless of location, and involves no cost [39].

### Experts

To remain consistent with the international aspect of the protocol, we will include a group of eight experts that consist of clinical and research experts in this field. The experts will be from Canada (*n* = 2), USA (*n* = 2), and The Netherlands (two locations, *n* = 4). The investigators (PM, JB, MR and JWG) discussed and confirmed a priori that each international location must consist of at least one clinical and one research expert. To be considered a clinical expert, the individual must have worked with adolescents and/or adults with CP for at least 5 years. To be considered a research expert, the individual must have published one or more articles related to an identified outcome of multimorbidity risk in this population (adolescents and/or adults with CP).

### Delphi survey

The initial stage of the Delphi survey will be a pre-round to obtain a list of OMIs that is as complete as possible. Based on the results from the literature searches performed by the two researchers, a list of studies with OMIs will be identified and divided into the seven defined outcomes: (1) physical behaviour, (2) nutrition, (3) sleep, (4) cardiorespiratory endurance, (5) body composition, (6) blood pressure and (7) lipids. Experts will be provided the results from the literature searches via e-mail, and requested to provide any additional OMIs that are relevant to an outcome but are not identified in the literature searches. These could include OMIs that are being used in clinical practice, ones used by a colleague, and/or ones that were read in an abstract or article or in a student’s thesis. Any proposed OMIs will be required to have a reference or abstract attached, to allow the two researchers to evaluate the quality of both the study and the OMI as per step 3 [30].

In round 1 of the Delphi survey, experts will receive an updated list of OMIs pertaining to each of the seven outcomes, which will be delivered by e-mail. The investigators will provide the experts with a spreadsheet consisting of seven tabs, one for each outcome. Every tab will include all associated OMIs that were obtained during step 2 and the Delphi pre-round, accompanied by a brief note of the methods/equipment used, a short description of the samples in which the OMI was used and graded evidence of the OMI resulting from step 3. A detailed description of the characteristics of each included study will be provided separately to assist experts in reviewing the OMIs. Study characteristics will include author and year of publication, the outcome(s) studied, the sample characteristics extracted in step 2 and the methodological quality of the study evaluated in step 3. Experts will be given detailed instructions and an instructional video outlining how to score each OMI on a 1–10 scale (1 = lowest, 10 = highest) for five different aspects of feasibility: applicability, patient feasibility, clinician feasibility, practical feasibility and overall rating. A comment box will be provided to allow experts the option to provide additional information to the researchers (i.e. explain responses or raise concerns), or to indicate that they are ignorant or uncertain. Experts will have 2 weeks to score the OMIs for feasibility. Reminder e-mails will be sent after 1 week and at 1 day before the end of the 2-week period. Mean scores will be calculated after receiving and aggregating the expert scores. OMIs with a mean overall score ≥ 7 will be retained, those with scores < 6 will be omitted and those with scores of 6–7 will be discussed among the investigators using the expert comments and quality scores. Moreover, we will examine differences between clinician and researcher scores and describe these results.

In subsequent rounds of the Delphi survey, experts will be presented the results from the previous round. All experts will see aggregated scores for each OMI, and a synopsis of the comments made by each expert (if applicable). Experts will be asked to consider the feedback (i.e. aggregated scores and comment synopsis) and again score the feasibility aspects for the remaining OMIs with an option to provide their rationale in a comment box. In these rounds, experts also will be asked to identify their preferred OMI for each outcome and to explain why. Similar to round 1, experts will have 2 weeks to score the OMIs for feasibility with reminder e-mails provided at the same time points. The expert scores will be processed in a similar fashion and extended with the preferred OMI scores. This process will continue until a single OMI per outcome is selected, based on ≥ 70% agreement among experts. The investigators will attempt to identify a provisional COS pertaining to the seven outcomes from the scores after two rounds. This will be based on aggregated scores (mean and median), expert opinion (i.e. rationales and additional information from the comments) and the quality of the studies and OMIs. The provisional COS will be presented to all experts, who will be asked whether they agree or disagree with the OMI for each outcome in the COS. If an expert disagrees with the suggested COS, they will be asked to provide their comments and reasons for disagreement [40]. From the decisions and comments made by the experts, a provisional COS will be presented and evaluated for final agreement. The COS will only become final after feasibility testing (phase 3) and stakeholder engagement (phase 4) (see below).

#### Step 5: patient/family judgement on relevance and completeness of selected outcomes

We will conduct a short online survey with patients with CP or families/caregivers of people with CP, to rate the importance of each outcome as something they would like their family doctor to measure and discuss with them.

#### Phase 3: feasibility study

##### Step 6: feasibility test of the COS in the target population

After developing a COS for multimorbidity risk assessment for use in clinical research and practice, the next stage will be to test the feasibility of the COS in a cohort of adolescents and adults with CP. Aspects of feasibility to be assessed from the perspectives of the clinicians and researchers will include ease of assessment, time required for completion and their confidence in the COS to assess multimorbidity risk. Time requirement and interpretation of results will be assessed (see below) to determine feasibility from the patient perspective.

### Participants

The feasibility study will focus on adolescents and adults aged 14 years and over, with a diagnosis of CP. We will include individuals with CP across all GMFCS levels (levels I–V), from three different international locations: Hamilton, ON, Canada; Ann Arbor, MI, USA and Rotterdam and Utrecht (combined), The Netherlands. The knowledge to be gained from this multinational study will be far superior to the minimal information that would be gained if we were to conduct the study at a single site, which has constrained the generalizability of research in this area [41].

### Recruitment strategy

Individuals with CP will be recruited during clinical visits to an adult rehabilitation centre or a child and youth clinic in Hamilton, ON, Canada; Ann Arbor, MI, USA and Rotterdam and Utrecht, The Netherlands. During clinical visits, a physician (JWG, EH or WS) or a study coordinator (PM or JB) from our research team will introduce the study to the patient, at which point the patient will have an opportunity to consent to participate in the study. Members of our team have used a similar recruitment strategy successfully in the past [26], and we are confident in achieving a total sample size of 75 (25 per geographical region) for testing the feasibility of the COS. As feasibility testing of the COS will be cross-sectional in nature, we will not include a control group at this time. We plan for a future grant application to fund an intervention study using the findings of our feasibility study, which will incorporate a control group.

### Sample size

An a priori criterion for success of this feasibility study is that a subsequent intervention trial would be feasible if the outcome variables were collected for ≥ 70% of participants. Using a 95% confidence interval (CI) for the proportion of eligible participants who complete the assessment, a margin of error of 0.05, a lower bound CI of 0.70 and an expected completion rate of 75%, the required sample for the feasibility study will be at least 75 participants. We aim to recruit five participants per GMFCS level per location (i.e. 5 participants * 5 GMFCS levels * 3 locations), for a total of 75 participants. As this is a cross-sectional study (i.e. single time commitment), we will not factor in the attrition rate.

### Assessments

Eligible participants who have provided written consent to participate in the feasibility study will be assessed. Participants will be invited to visit the relevant setting, in which we will execute the OMIs selected for the COS. Assessments will be conducted by the clinicians and researchers involved, where applicable with support from research/laboratory assistants. Based on the outcomes that have been identified in step 1, we estimate that it will take 3–4 h to conduct the total set of OMIs. Naturally, the collected data will provide insight into the risk profile of the individual participant. We hope that the measures will be integrated into clinical care as much as possible. It is conceivable that the total data collection time may be spread over a single assessment or multiple assessments (e.g. body composition and blood pressure measurements in clinic, but an additional session for measurement of cardiorespiratory endurance). The total time required will be explored in our feasibility study. The COS will be qualitatively evaluated to examine its acceptability as a whole, by both the participants and clinicians/researchers. After the measurements the participants will be asked about their experience of the COS, including time required to complete the assessments, via a short survey. Upon completion of all measurements, we will question the clinicians and researchers who conducted the assessments about the ease of assessment, completion time and their confidence in the COS, also via a short survey. Together with the collected data, the feedback from the participants, clinicians and researchers will provide a clear indication of the feasibility of the COS for future use in clinical research and practice.

#### Phase 4: patient and family/caregiver engagement

##### Step 7: final agreement on the COS among stakeholders

As a final step and after taking into consideration the qualitative evaluations from study participants, clinicians and researchers, we will organize a face-to-face stakeholder meeting to reach final agreement on the COS. Adolescents and adults with CP and their families and/or caregivers, as stakeholders in this project, will be recruited with support from the American Academy for Cerebral Palsy and Developmental Medicine (AACPDM) family/participant education forum. The AACPDM education forum is held annually at the AACPDM conference. Prior to the meeting, we will work with the AACPDM administrative leaders to have an advertisement positioned on their website asking for adolescents and adults with CP (and their families/caregivers) to participate in a meeting to help review and finalize a COS for multimorbidity risk. We will invite four adolescents and four adults with CP of varying GMFCS levels, who did not participate in the feasibility study, and their families and/or caregivers (if applicable), to take part in the meeting, which will occur during the AACPDM 2018 conference (9–13 October 2018).

#### Dissemination

Details of the finalized COS will be disseminated through publication in a scientific journal, presentation(s) at international scientific conferences and research rounds at clinics and academic institutions at each international location.

## Discussion

The aim of this project is to develop and test the feasibility of a COS to assess multimorbidity risk in adolescents and adults with CP. Ultimately, the COS will be used to understand, treat and prevent multimorbidity in this population, while being utilized in a clinical and/or research setting. The development of this COS is expected to have the potential to be generalized to other types of child-onset neurodevelopmental disabilities.

A strength of the proposed work is the inclusion of clinical and research experts in this field (COS development) and individuals with CP (feasibility study), from international locations. The knowledge to be gained from an international study will be significant and meets a major limitation in multimorbidity risk research in this population (i.e. studies of small sample sizes that are geographically isolated). If we are able to conduct the feasibility study successfully and receive positive feedback from individuals with CP, their families/caregivers and clinicians and researchers, our next step will be to apply for funding to conduct an intervention study in this population aiming to prevent multimorbidity risk, including other geographic locations worldwide. In the meantime, the development and feasibility testing of a COS for adolescents and adults with CP will improve the consistency of CP research moving forward. Ultimately, we aim to utilize the COS in clinic to work towards developing a database that will allow for harmonization of data and the ability to document changes over time, which will enhance and accelerate our understanding of multimorbidity risk and presentation in this population, and will help to overcome the issues of current small-scale studies. As well, performing the COS assessment in clinic will allow us to obtain a risk profile for the patient, which can help inform an individualized treatment plan.

A challenge we faced with this protocol was selecting when to engage individuals with CP and their families/caregivers as key stakeholders in COS development. Ideally, we would have included these stakeholders throughout the study from the very beginning to the end. Despite not including individuals with CP in phase 1 of the project, we believe that the outcomes we selected align with the top research priorities identified in part by individuals with CP and their families: understanding how to prevent secondary impairments related to aging with CP and identifying effective long-term exercise strategies to improve activity and health across their lifespan [42]. Moreover, in addition to the Delphi survey among professionals, we will also conduct an online patient/family survey to inform us whether the target population agrees that the outcomes to measure are relevant. Due to the focus on knowledge synthesis in the Delphi survey with research rigor and the terminology involved in quality assessments of studies and OMIs, we decided it would be more pragmatic to develop a provisional COS amongst clinicians and researchers, and then bifurcate to feasibility testing and a stakeholder meeting, to incorporate perspectives from individuals with CP and their families/caregivers and come to a final agreement on the COS. A former study that attempted to develop a COS with patient perspectives from the onset of the idea was reported as challenging [43].

Despite an effort to include expert clinicians and researchers working with adolescents and adults with CP who are knowledgeable of multimorbidity, none of these individuals considered themselves as experts in the outcomes of nutrition and sleep in CP. This identifies an important gap in clinical research in this population; if nutrition and sleep are to be considered important components of multimorbidity risk prevention in people with CP [44], clinicians and researchers need to be trained in these outcomes in order to assess and manage these components of health.

### Trial status

At the time of submission, we have included experts for the Delphi survey. Recruitment for the feasibility study will start in June 2018 subject to the ethics approval from all institutions involved.

## Additional files

Additional file 1: Complete search strategy for Embase. (DOCX 15 kb)

Additional file 2: Article screening form including inclusion/exclusion criteria. (DOCX 16 kb)

## Abbreviations

AACPDM: American Academy for Cerebral Palsy and Developmental Medicine
CDE: Common data elements
COMET: Core Outcome Measures In Effectiveness Trials
COS: Core outcome set
COSMIN: Consensus-based standards for the selection of health measurement INstruments
CP: Cerebral palsy
GMFCS: Gross Motor Function Classification System
OMI: Outcome measurement instrument
