# Supplementary material for: Multimorbidity risk assessment in adolescents and adults with cerebral palsy: a protocol for establishing a core outcome set for clinical research and practice
Source: Trials. 2019 Mar 19;20:176. doi: 10.1186/s13063-019-3265-z (PMC6425572; doi:10.1186/s13063-019-3265-z)
Supplement: Supplementary file 1 — Complete search strategy for Embase. (DOCX 15 kb) [file 13063_2019_3265_MOESM1_ESM.docx]

**Additional file 1**

**Complete search strategy for Embase**

*Population of interest: adolescents and adults with cerebral palsy*

('cerebral palsy'/exp/mj OR (((cerebral* OR brain OR spastic) NEXT/2 (pals* OR paralys* OR hemipleg* OR diplegi* OR paresis)) OR 'encephalopathia infantilis'):ab,ti) AND (('adolescence'/exp OR 'adolescent'/de OR (teenage* OR puberty):ab,ti) OR ('adult'/exp OR (adult* OR elderly OR older OR aging OR pensioner OR retiree OR (grow* NEXT/1 up*)):ab,ti))

AND

*Construct of interest (2,3): body size and body composition*

('anthropometric parameters'/exp OR 'body composition'/exp OR obesity/exp OR ‘BMI (body mass index)’ OR ‘body mass index’ OR ‘Quetelet index’ OR adiposit* OR obesit* OR overweight OR ((body) NEAR/1 (height OR mass OR size OR composition OR fat OR lipid)) OR ((fat OR adipose) NEAR/2 (distribution OR mass OR tissue)) OR ((waist OR hip OR waist-hip) NEAR/1 (circumference OR ratio)):ab,ti)

OR

*Construct of interest (4): physical behavior*

('physical activity'/exp OR 'physical inactivity'/exp OR 'sedentary lifestyle'/exp OR sitting/exp OR 'energy expenditure'/exp OR 'motor activity'/de OR accelerometer/de OR accelerometry/de OR actimetry/de OR ((sedentary OR physical OR motor) NEAR/1 (lifestyle OR activit* OR inactivity)) OR ((caloric OR energy) NEAR/1 (expenditure)) OR sitting OR acceleromet* OR actimetry OR actigraph* OR ((activity) NEAR/1 (monitor OR tracker)):ab,ti)

OR

*Construct of interest (5): sleep*

(sleep/exp OR 'sleep disorder'/exp OR sleep* OR dream* OR dyssomnia* OR hyposomnia* OR hypersomnia* OR insomnia* OR parasomnia* OR sleeplessness OR sleepiness OR somnolence OR tiredness OR tired OR ((sleep) NEAR/2 (disorder* OR disturbance OR paralysis)):ab,ti)

OR

*Construct of interest (6): nutrition*

(nutrition/exp OR 'feeding behavior'/exp OR 'dietary intake'/exp OR feeding OR nutrition OR diet OR dieting OR ((diet* OR nutrition*) NEAR/1 (survey* OR assessment OR state OR status)) OR ((food OR feed OR energy OR calor* OR dietary OR nutrient) NEAR/1 (uptake OR intake OR consumption)) OR ((feeding OR eating OR drinking OR alimentary OR nutrition*) NEAR/1 (behavior* OR habit* OR pattern*)):ab,ti)

OR

*Construct of interest (7): blood pressure*

('blood pressure'/exp OR 'blood pressure monitoring'/exp OR 'abnormal blood pressure'/exp OR 'blood pressure measurement'/de OR 'hypertension encephalopathy'/de OR 'blood pressure monitor'/exp OR (((blood OR vessel* OR vascul* OR intravascul* OR venous OR arter*) NEAR/3 (pressure OR tension*)) OR hypotens* OR hypertens* OR prehypertens* OR normotens*):ab,ti)

OR

*Construct of interest (8): blood lipids*

(dyslipidemia/exp OR hyperlipidemia/exp OR hypolipemia/exp OR cholesterol/exp OR triacylglycerol/de OR dyslipid* OR hyperlipid* OR hypolip* OR cholester* OR triacylglycerol OR triglyceride* OR ‘lipid profile’:ab,ti)

NOT

*Limitations*

([animals]/lim NOT [humans]/lim) NOT ([Conference Abstract]/lim OR [Letter]/lim OR [Note]/lim OR [Editorial]/lim)

Note:

Construct of interest (1) cardiorespiratory endurance was not included in the search strategy, since an existing, up-to-date systematic review was already identified. The search strategy was translated for MEDLINE/Ovid, MEDLINE/Pubmed, and PsychINFO.
