# Supplementary material for: Multimorbidity risk assessment in adolescents and adults with cerebral palsy: a protocol for establishing a core outcome set for clinical research and practice
Source: Trials. 2019 Mar 19;20:176. doi: 10.1186/s13063-019-3265-z (PMC6425572; doi:10.1186/s13063-019-3265-z)
Supplement: Supplementary file 2 — Article screening form including inclusion/exclusion criteria. (DOCX 16 kb) [file 13063_2019_3265_MOESM2_ESM.docx]

**Additional file 2**

**Article screening form including inclusion/exclusion criteria**

Articles answered “yes” or “unclear” to *all criteria* should be included.

**1. Is this article about humans?**

If no 🡪 EXCLUDE

If yes or unclear 🡪 go to next question

**2. Is this article about people with cerebral palsy?**

If no 🡪 EXCLUDE

If yes or unclear 🡪 go to next question

**3. Is this article about adolescents (≥14years of age) or adults (≥18years of age)?**

If no 🡪 EXCLUDE

If yes or unclear 🡪 go to next question

**4. Is this article about…**

**Physical activity, physical inactivity, sedentary lifestyle, sitting, energy expenditure, motor activity, accelerometry**

**OR**

**Anthropometric parameters, body composition, BMI, adiposity, obesity, overweight, height, fat distribution, adipose distribution, waist circumference, hip circumference, waist-to-hip ratio**

**OR**

**Sleep, sleep disorder, dyssomnia, hyposomnia, hypersomnia, insomnia, parasomnia, sleeplessness, sleepiness, tiredness, sleep disturbance, sleep paralysis**

**OR**

**Blood pressure, blood pressure monitoring, abnormal blood pressure, blood pressure measurement, hypertension, blood pressure monitor, artery pressure, hypotension, hypertension, prehypertension, normotension**

**OR**

**Dyslipidemia, hyperlipidemia, hypolipemia, cholesterol, triacylglycerol, lipid profile**

**OR**

**Nutrition, feeding behavior, dietary intake, feeding, diet, diet survey, nutrition survey, diet assessment, nutrition assessment, diet state, nutrition state, diet status, nutrition status, food, feed, energy, calorie, nutrient, food uptake, food intake, food consumption, energy uptake, energy intake, energy consumption, calorie uptake, calorie intake, calorie consumption, dietary uptake, dietary intake, dietary consumption, nutrient uptake, nutrient intake, nutrient consumption, feeding behavior, feeding habit, feeding pattern, eating behavior, eating habit, eating pattern, drinking behavior, drinking habit, drinking pattern, alimentary behavior, alimentary habit, alimentary pattern, nutrition behavior, nutrition habit, nutrition pattern**

If no 🡪 EXCLUDE

If yes or unclear 🡪 go to next question

**5. Is this study a randomized controlled trial, intervention study, longitudinal study or observational / cross-sectional study?**

If no 🡪 EXCLUDE

If yes or unclear 🡪 include
